# Metabolic and Evolutionary Engineering of Diploid Yeast for the Production of First- and Second-Generation Ethanol

**DOI:** 10.3389/fbioe.2021.835928

**Published:** 2022-01-28

**Authors:** Yang Sun, Meilin Kong, Xiaowei Li, Qi Li, Qian Xue, Junyan Hou, Zefang Jia, Zhipeng Lei, Wei Xiao, Shuobo Shi, Limin Cao

**Affiliations:** ^1^ Key Laboratory of Straw Comprehensive Utilization and Black Soil Conservation, Ministry of Education, College of Life Science, Jilin Agricultural University, Changchun, China; ^2^ College of Life Sciences, Capital Normal University, Beijing, China; ^3^ Beijing Advanced Innovation Center for Soft Matter Science and Engineering, College of Life Science and Technology, Beijing University of Chemical Technology, Beijing, China

**Keywords:** *Saccharomyces cerevisiae*, 1G and 2G ethanol, xylose, evolutionary engineering, lignocellulosic hydrolysates

## Abstract

Despite a growing preference for second-generation (2G) ethanol in industries, its application is severely restricted owing to a major obstacle of developing a suitable yeast strain for fermentation using feedstock biomasses. In this study, a yeast strain, *Saccharomyces cerevisiae* A31Z, for 2G bioethanol production was developed from an industrial strain, Angel, using metabolic engineering by the incorporation of gene clusters involved in the xylose metabolism combined with adaptive evolution for evolving its anti-inhibitory properties. This strain outcompeted its ancestors in xylose utilization and subsequent ethanol production and manifested higher tolerance against common inhibitors from lignocellulosic hydrolysates, and also it lowered the production of glycerol by-product. Furthermore, A31Z outperformed in ethanol production using industrial hydrolysate from dried distillers grains with solubles and whole corn. Overall, this study provided a promising path for improving 2G bioethanol production in industries using *S. cerevisiae*.

## Introduction

Second-generation (2G) ethanol produced from lignocellulosic biomasses has received growing attention and has been considered as a promising replacement for petroleum fuel due to its eco-friendly features (Antunes et al., 2019); and lignocellulosic wastes are recognized as the most feasible feedstock used for the 2G ethanol production owing to their low costs, renewability, and availability (Fatma et al., 2018). In the process of feedstock utilization, lignocellulosic biomasses were initially subjected to break the rigid structure of lignin, hemicelluloses, and cellulose, followed by hydrolyzation with enzymes or chemicals. Different mono-sugars could be released from the hydrolysate, such as glucose, xylose, and arabinose, which would be converted into ethanol and other bioproducts by microbes. The brewer’s yeast *Saccharomyces cerevisiae* is one of the most widely used cell factories. However, *S. cerevisiae* cannot assimilate xylose naturally, resulting in incomplete usage of sugars in lignocellulosic hydrolysates. This is one of the problems for inefficient production of 2G ethanol. Recently, various strategies have been attempted to develop a modified yeast strain so that it can maximize the ethanol production using xylose as the substrate (Zhang et al., 2019).

The metabolism of xylose in yeast has been extensively studied (Li X. et al., 2019). Generally, nice enzymes, including XR, XDH, Xks1p, Tal1p, Pyk1p, Rki1p, Rpe1p, Tkl1p, and MGT05196, play a key role in xylose utilization in yeasts. Xylose reductase (*XR* or Xy11), encoded by the *XR* gene, was used to convert xylose into xylitol, which was then transformed into xylulose using xylitol dehydrogenase (*XDH*, Xyl2). Subsequently, xylulose was phosphorylated to form xylulose-5-phosphate by xylulokinase (*XK*, Xks1) in the pentose phosphate pathway (PPP) (Li X. et al., 2019). The implementation of this three-enzyme pathway (XR, XDH, and XK) enabled the cell growth and production of ethanol from xylose. On the other hand, xylose could be also converted into xylulose directly by xylose isomerase (XI). The transaldolase Tal1, responsible for the regulation of the balance of metabolites in the pentose phosphate pathway, and the pyruvate kinase Pyk1, involved in the conversion of phosphoenolpyruvate and ADP to pyruvate and ATP in glycolysis, are usually considered rate-limiting enzymes in the non-oxidative pentose phosphate and glycolysis pathways, respectively. Variations in the expression of these two enzymes would result in the fluctuation of ethanol metabolism notably (Zhang et al., 2019). Upregulation of the gene *PYK1* promoted the pyruvate metabolic flow to the ethanol production (Gruning et al., 2011; Cao et al., 2014; Gruning et al., 2014; Xu et al., 2016). Finally, the transporter Mgt05196, responsible for the uptake of xylose in the absence of glucose inhibition, was crucial for simultaneously catabolizing glucose and xylose in yeast (Wang et al., 2015). Currently, the most efficient xylose-fermenting yeast strains could use both glucose and xylose as substrates from lignocellulosic feedstocks and could generate ethanol yields of 0.46–0.47 g/g (Zhang et al., 2019). Most yeasts developed so far for 2G ethanol production are haploids due to their ease with genetic manipulations. Nevertheless, diploid yeasts or polyploids have shown to be more resistant to harsh environmental conditions in industries, whereas the expertise from haploid yeasts facilitated paving a practical path for constructing the diploid yeast strain for bioethanol production in this study.

Owing to the recalcitrant structure of lignocellulose biomass, pretreatments are needed prior to the hydrolysis to facilitate the following sugar conversion by microbes (Baruah et al., 2018; Chandel et al., 2018; Abo et al., 2019; Rempel et al., 2019). Various strategies have been adopted, which are typically categorized into four types: physical, chemical, physicochemical, and biological approaches (Baruah et al., 2018). Chemical and enzymic degradation have gained much interest in recent years due to their high hydrolytic biomasses and low costs (Wang et al., 2017; Tian et al., 2018). Subsequently, polysaccharides produced in these ways could readily be transformed into monosaccharides with high efficiency and specificity, especially by using enzymes (Wu et al., 2019). The hydrolysates from lignocellulosic feedstocks such as corn stover (CS), corn cob (CC), or the specific four pairs of *Miscanthus* were widely used for bioethanol production (Alam et al., 2019; Watanabe et al., 2019; Wu et al., 2019). However, various inhibitory substances, including acetate, furans, and phenolic compounds, produced during the hydrolytic process severely suppressed the growth of yeasts, leading to the poor performance of subsequent fermentation (Klinke et al., 2004; Koppram and Olsson, 2014; Gutierrez-Macias and Nacheva, 2015). To address these problems, various studies revealed that yeast cells could develop high tolerance against the inhibitory effects of lignocellulosic hydrolysates by adapting them in a medium containing external acetate, which led to a pronounced improvement of the xylose utilization and therefore ethanol production (Li H. et al., 2019; Kwak et al., 2019; Zhang et al., 2019). Deparis et al. (2017) developed yeasts with high resistance to the inhibitors by repetitive batch cultivation of the strains in lignocellulosic hydrolysate. *In situ* detoxification of fural was confirmed to be one of the underlying mechanisms for tolerance to phenolic compounds in yeasts (Deparis et al., 2017). In addition, the enzymes Pad1 and Fdc1, responsible for aromatic acid conversion, were shown to be involved in the removal of fural from hydrolysates of lignin (Scanes et al., 1998).

In this study, based on our previous study in haploid yeasts (Zhang et al., 2019), this work constructed several diploid ethanol-producing yeasts that can use xylose by incorporation of various metabolic gene clusters into the genome of two industrial diploid yeasts as well as direct adaption. The best diploid producer A21Z showed higher fermentation efficiency compared to its ancestors. Then it was further optimized by adaptation of A21Z in medium containing 15% of industrial hydrolysate from wheat straw, evolving into strain A31Z which displayed a superior xylose utilization and tolerance against inhibitors from hydrolysates of lignocelluloses compared to its ancestors. Our study showed that metabolic engineering combined with adaptive evolution was suitable for improving yeast resistance to inhibitory conditions and enhancing the xylose utilization for ethanol production in industries.

## Materials and Methods

### Construction of Yeast Strains

All DNA manipulations were carried out in *Escherichia coli* strain DH5α as described (Sambrock and Russel, 2001). *S. cerevisiae* diploid strains, Angel and Henderson, used in this study were obtained from *Angel Yeast Co*., *Ltd.* Plasmid pUC-TTRR (Xiong et al., 2011) containing the non-oxidative phosphate pathway with four gene transcription units *PDC1p-TKL1*-TKL1t/*PGK1p-TAL1*-TAL1t*/TPI1p-RKI1*-RKI1t*/ADH1p-RPE1*-RPE1t and pUC-fps1-nat (Zhang et al., 2018) containing xylose utilization pathway with three gene transcription units *ADH1p*-XYL1-ADH1t/*PGK1p*-XYL2-PGK1t/*PGK1p*-XKS1-PGK1t were used for genetic engineering in diploid Angel and Henderson, respectively, resulting in strain ABN and BBN, separately. Genes (*TAL1*, *TKL1*, *RKI1*, and *RPE1*) in the non-oxidative phosphate pathway for plasmid pUC-TTRR were integrated into the non-functional sites *HOG1* and *HOG2*. This pUC-fps1-nat was used to integrate the three genes of XYL1, XYL2, and XKS1 into the yeast chromosome site *FPS1*. Repeated batch cultivation of yeast strain ABN was performed for independent evolution using a previously reported method (Zhang et al., 2019). Serial transfer was done by alternating cultivation in YP with 20 g/L xylose and 3 g/L to 12 g/L acetate. At periodic intervals (5 days), the fastest growing colonies were selected for independent evolution to propagate the improved fitness colonies. After one and half year, the evolved strain A1 was obtained from the wild-type strain ABN. Then the new evolved strain A2 was further obtained from the aforementioned strain A1. Thus, strains A1 and A2 were obtained after a 3-year evolutionary process. The strain A21Z was obtained by integrating the expression cassette 1z-e7 (*XYL1(K270R)-XYL2-TAL1-PYK1-MGT05196-PYK1-MGT05196*) (Zhang et al., 2019) into the strain A2. And the strain A22Z was obtained by integrating another copy of the 1z-e7 into A21Z. Then the strain A31Z was obtained by adapting growth of A21Z in 15% wheat straw stover hydrolysate using a previously reported method (Zhang et al., 2019). *S. cerevisiae* strains used in this study are listed in Supplementary Table S1. The transformation of yeast strains in this study was done following the standard LiAc/SS carrier DNA/PEG method (Gietz and Schiestl, 2007).

### Medium

The strains cultured in YPD (20 g/L tryptone, 10 g/L yeast extract, and 20 g/L glucose) at 200 rpm for 24 h were collected, centrifuged, and washed, which were then inoculated into a 500-ml shaker flask (initial OD_600_ of 1) containing fresh fermentation medium. The YP medium contained 50 g/L glucose, 50 g/L xylose, and 3 g/L acetate at pH 5.5 for the comparative fermentation for Angel, Henderson, ABN, and BBN. The YP medium of mimic Dried distillers grains with solubles (DDGS) hydrolysate contained 100 g/L glucose and 50 g/L xylose at pH 5.5 for fermentation performance analysis of A31Z. The evolutionary engineering of strains were conducted in YP + acetate + xylose domestication medium (YPAX) with 10 g/L yeast extract, 20 g/L tryptone, 20 g/L xylose, and 8 g/L of acetate.

### Enzymatic Hydrolysis of Lignocellulosic Biomasses

The biomass powders (0.300 g) of *Miscanthus*, maize, and wheat straw, were, respectively, incubated with 0.012 g mixed cellulases (cellulases at 10.60 FPU g^−1^ biomass and xylanase at 6.72 U g^−1^ biomass from Imperial Jade Bio-technology Co., Ltd) containing 0.8% Tween-80 at 5% solid loading, and shaken under 150 rpm for 48 h at 50°C. The samples were centrifuged at 3,000 g for 5 min, and the supernatants were then collected to conduct hexose and pentose assay. The sugar yields (% dry matter) released from enzymatic hydrolysis of *Miscanthus*, maize, and wheat straw with different solid-to-liquid ratio fermentation was described in Supplementary Table S2. The cell wall composition (% dry matter) from enzymatic hydrolysis of *Miscanthus*, maize, and wheat straw is described in Supplementary Table S3.

### Fermentation From Hydrolysis of Corn Starch and Dried Distillers Grains With Soluble

To obtain hydrolysis of corn starch, starch samples with 30% solids were mixed with the enzyme liquozyme at a loading of 0.064% under 85°C with a pH at 5.7 for 4 h. Then the saccharification was performed by the addition of 0.1% starch hydrolyzing enzyme and 0.6% Novozymes Celluclast^®^ at pH 4.8 for 0.5 h. The fermentation was carried out under 30°C at pH 4.6 and 150 rpm for 72 h. The initial value of OD_600_ nm for the fermentation was set up as 1.0. The residual corn starch was recovered with 1% H_2_SO_4_ under 95°C for 90 min upon finishing based on the situ dilute acid pretreatment. Then the sample was subjected to evaporation to remove the remaining ethanol at 0.09 mpa under 85°C for 30 min. After this, water was added to the sample up to its initial volume accompanied with the adjustment of the pH at between 4.8 and 5.0. Subsequently, 0.6% cellulases and 1.5% xylanase were added and incubated at 50°C, 250 rpm for 24 h. Finally, the hydrolysis was adjusted to pH 4.6 and adopted as the medium for fermentation from DDGS.

### Simultaneous Saccharification and Co-fermentation of Whole Corn

The corn stover was pretreated using dilute sulfuric acid (1% v/v) at 160°C for 10 min with a solid loading at 10% (w/v). Then the hemicelluloses released from the corn stover were incubated under 50°C for 48 h at 250 rpm mixed with 40 mg cellulases per gram glucan (containing cellulases and xylanase with a ratio at 9:1). Meanwhile, the corn flour underwent liquefaction using the liquozyme at 0.064% under 85°C for 4 h.

The schematic process of SSCF is illustrated in Supplementary Figure S1. The co-fermentation was performed under 30°C for 72 h containing 12% corn flour and 12% corn stover hydrolysis, and 0.1% diastase with an initial OD_600_ of 1.0.

### Analytical Methods

The measurements of OD_600_ nm and cell dry weights (CDW) were recorded according to the previous description (Cao et al., 2014). Glucose, xylose, xylitol, ethanol, and acetic acid were analyzed by HPLC using its related column and detector run with 5 mM H_2_SO_4_ as the mobile phase at a flow rate of 0.4 ml/min (Cao et al., 2014). The pentose and hexose of soluble sugars were, respectively, detected as described previously (Wu et al., 2019).

### Calculation of Ethanol Production

The following formula was adopted for the calculation of ethanol production:

$$Ethanol\;yield=\frac{Ethanol\;concertration\;at\;the\;end\;of\;fermentation}{Total\;sugars\;concertration\;at\;0h\;}.$$

## Results

### Fermentation Performance of Original Yeast Strains, Angel, and Henderson

**FIGURE 1:**
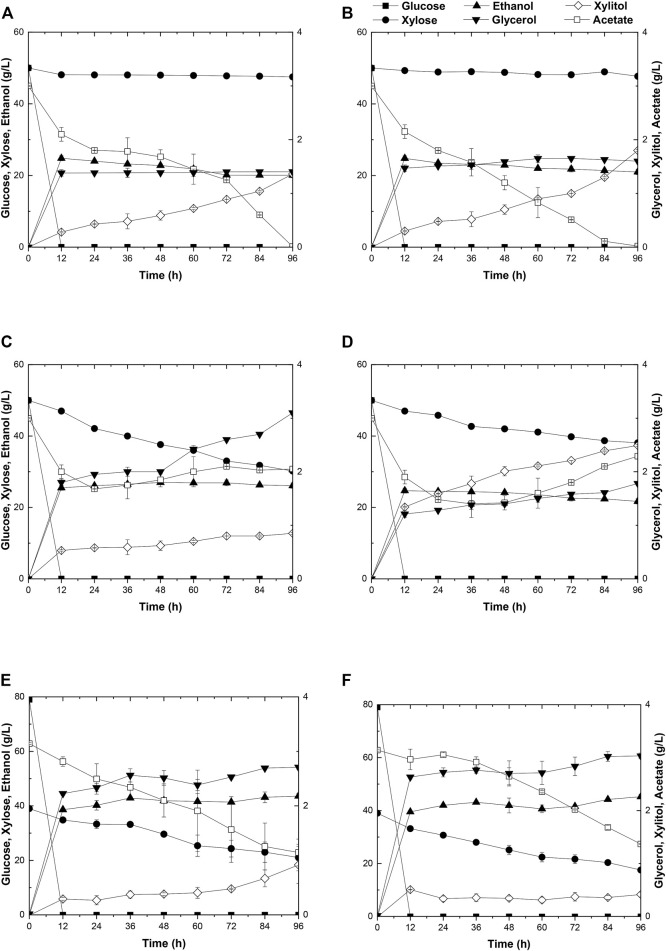
Time-dependent ethanol fermentation of yeast strains in this study. Fermentation profile of yeast Angel **(A)** and yeast Henderson **(B)** in mixed sugar medium of 50 g/L glucose and 50 g/L xylose containing 3 g/L acetate. Fermentation of ABN **(C)** and BBN **(D)** in mixed sugar medium of 50 g/L glucose and 50 g/L xylose containing 3 g/L acetate. Fermentation profile of A1 **(E)** and A2 **(F)** in YP medium containing 3 g/L acetate, 80 g/L glucose, and 40 g/L xylose. Each experiment was repeated three times, and two parallel controls were set at the same time.

Due to the better performance of diploid yeast strains in cell viability, genetic stability, and stress endurance than those of haploid strain in the fermentation process (Kim et al., 2013), two industrial diploid yeasts from two individual ethanol companies, *S. cerevisiae* Angel and Henderson, were selected as the starting strains for the following genetic modification and evolution. As expected, neither of the two yeasts could consume xylose. During a 96-h fermentation in the medium containing 50 g/L glucose, 50 g/L xylose, and 3 g/L acetate, both strains consumed all of the glucose within the initial 12 h, and ethanol productions reached the maximum of 27.4 g/L for Angel and 24.8 g/L for Henderson(Figures 1A,B). Henderson formed a slightly higher level of xylitol in this process, with 1.80 g/L xylitol production, whereas 1.36 g/L was produced by strain Angel. No significant difference in glycerol accumulation was observed, and both strains exhibited high capabilities of acetate consumption, with 2.3 and 1.2 g/L residue within 48 h, respectively.

### Construction of Diploid Xylose Assimilating Yeast Strains ABN and BBN and Their Fermentation Analysis

To enhance xylose utilization in yeast, diploid xylose assimilating yeast strains ABN and BBN were constructed by incorporating the two sets of gene clusters involved with xylose metabolism *XYL1/XYL2/XKS1* and T*AL1/TKL1/RKI1/RPE1* into the strains of Angel and Henderson, respectively. The fermentation performance of these strains was evaluated by growing them separately in a medium added with mixed sugars and external acetate, same as before. Both strains consumed all of glucose in the medium within 12 h. In addition, strain ABN converted 50 g/L glucose and 21.2 g/L xylose into 27.4 g/L ethanol in 96 h, whereas BBN consumed 50 g/L glucose and 10.9 g/L xylose yielding 25.0 g/L ethanol within the same duration (Figures 1C,D). Compared to BBN, strain ABN displayed better xylose metabolic efficiency with a similar glycerol accumulation and lower xylitol accumulation (Figures 1C,D). Therefore, ABN was selected for further evolutionary engineering. Unexpectedly, the acetate utilization of these two engineered strains reduced significantly compared to those of their original strains, indicating that the metabolism of acetate was inhibited after the introduction of the xylose metabolic pathway.

### The Generation of Strain A21Z and Fermentation Performances of This Strain and Its Ancestors A1 and A2

Toxic substances are always accumulated in the hydrolysates of lignocellulosic materials to some extent, which brings about great hindrance to the cell growth of yeasts due to the intracellular acidification, resulting in poor xylose utilization and 2G ethanol formation. Two approaches, by acetate catabolism or by mechanisms of acetate tolerance, were prompted to counteract the negative effects of acetate in the media (Zhang et al., 2019). Here, adaptive evolution was adopted to increase the metabolic efficiency of mixed sugars in the presence of acetate. Initially, the diploid strain ABN was subjected to adaptive evolution, resulting in strain A1 in YPAX domestication medium using a previous method (Zhang et al., 2019). Then an A2 strain was obtained by growing strain A1 in the YPAX medium for another round of adaptation. Fermentation of A1 and A2 was separately performed in the medium containing 80 g/L glucose, 40 g/L xylose, and 3 g/L acetate (Figures 1E,F). Both strains A1 and A2 showed significantly higher xylose consumption capacities than ABN. They consumed the entire glucose in the medium within 12 h, leading to ethanol yields of 88.1 and 88.2% of the theoretical values (0.51 g ethanol/g total sugars), respectively (Tran et al., 2020). Compared with strain ABN, strain A1 totally yielded 43.6 g/L of ethanol by consumption of 80 g/L glucose and 17.9 g/L more of xylose, and 45.2 g/L from A2 finally by consumption of 80 g/L glucose and an additional 21.4 g/L of xylose. Furthermore, A1 and A2 displayed increased acetate consumptions compared to ABN.

**FIGURE 2:**
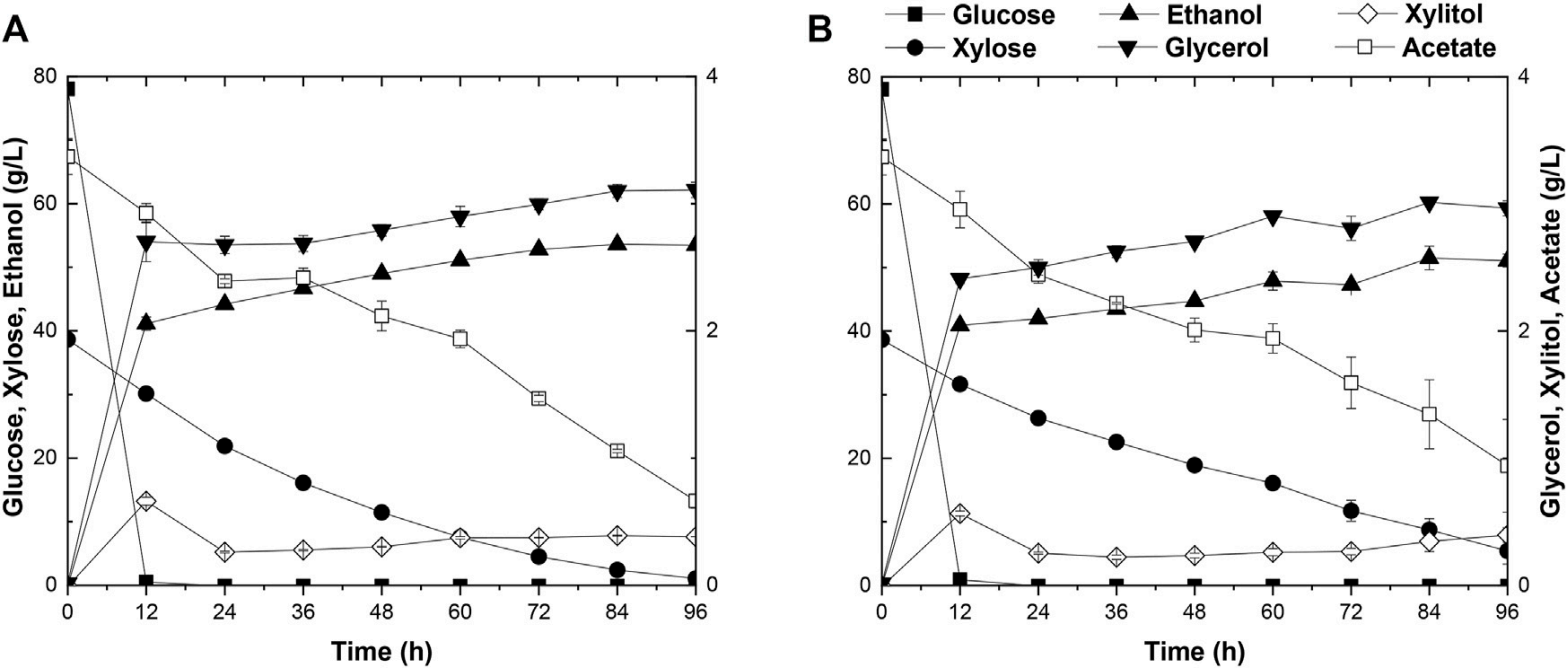
Time-dependent ethanol fermentation of strains A21Z and A22Z in YP medium. Fermentation profile of A21Z **(A)** and A22Z **(B)** in YP medium with 3 g/L acetate, 80 g/L glucose, and 40 g/L xylose.

Strain A2 consumed a 3.5 g/L more xylose with a 1.6 g/L higher ethanol production than strain A1. The superior performance of this strain may likely lie in the more favorable mutations accumulated during the two-round evolutionary engineering in this strain (Figures 1E,F). A2 was then selected for subsequent engineering. To further improve the xylose metabolism, the six gene clusters, 1z-e7, (*XYL1(K270R)-XYL2-TAL1-PYK1-MGT05196-PYK1-MGT05196*) with the characteristics of redox balance, and 2z-e7, (*XYL1 (K270R) - XYL2-TAL1-klPYK1-MGT05196-klPYK1-MGT05196*) with an exogenetic gene *PYK1* from *Kluyveromyces lactis* were introduced into the strain A2 to obtain strain A21Z and A22Z, respectively, according to previous studies (Zhang et al., 2019). After 96 h, the xylose consumption and ethanol production of A21Z were shown to be 37.5 g/L and 53.5 g/L (Figure 2A), respectively, reaching a yield of 87.4% of the theoretical ethanol production, whereas 33.2 g/L and 51.1 g/L for the strain A22Z were observed (Figure 2B), respectively, with a 83.5% theoretical yield of ethanol production. Within 96 h, most xylose in the medium (with 1.1 g/L xylose remained) was consumed by strain A21Z, producing 8.3 g/L more ethanol than that of A2. Overall, A21Z showed a superior performance in xylose consumption, ethanol yield, and sugar-to-alcohol conversion (Figures 2A,B) compared with its ancestors and A22Z, which therefore encouraged using this strain for the subsequent evolutionary engineering further.

### A31Z Strain Evolved by Adapting Strain A21Z in the Hydrolysate of Wheat Straw Stover With 15% Solid Loading

**FIGURE 3:**
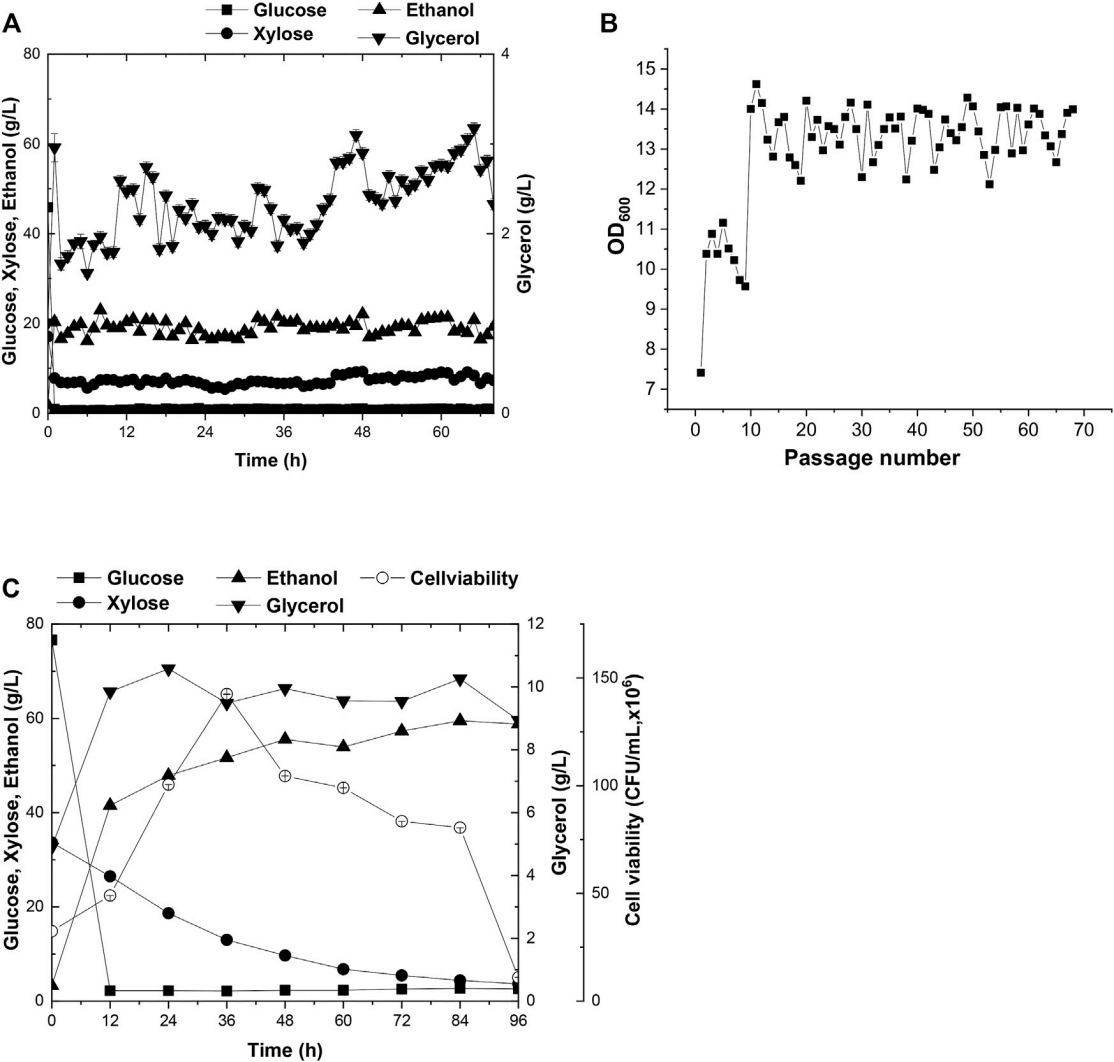
Time-dependent fermentation of A21Z and A31Z. Fermentation profile of A21Z in treated wheat straw hydrolysate with 45.85 g/L glucose, 17.07 g/L xylose, 1.84 g/L ethanol, and 1.01 g/L glycerol **(A,B)**. SSCF profile of A31Z **(C)**.

Currently, wheat straw is the most abundant lignocellulosic biomasses among agricultural residues. However, intractable compositions accumulated in the hydrolysates, such as acetate, severely impede the cell growth in the subsequent fermentation procedures. To overcome these difficulties, adaptive evolution was broadly employed to customize a target strain in stressful industrial pretreated products (Zhang et al., 2019). In this study, solid wheat straw stover pretreated product was added to the cultural medium at a 15% (w/v) proportion for domestication of A21Z by serial passages (Figures 3A,B). The seed culture of A21Z was inoculated into the wheat straw pretreated products at 10.0% (v/v); the initial concentration of glucose, xylose, ethanol, and glycerol in the medium for evolution was 45.85 g/L, 17.07 g/L, 1.84 g/L, and 1.01 g/L, respectively. Yeast cells initially underwent a lag in growth upon inoculation in the fresh wheat straw–pretreated products medium and did not manifest efficient utilization of the pretreated products immediately for ethanol production. After passages in the medium, the strain A31Z was evolved, displaying robust growth in the medium (Figure 3B), even though the xylose consumption and ethanol production did not show significant increases across the passages, which suggested that the strain A31Z had adapted to the cultural medium, and could proceed to the simultaneous saccharification and co-fermentation (SSCF).

### Simultaneous Saccharification and Co-Fermentation of Strain A31Z Using Wheat Straw

Prior to SSCF, the wheat straw was subjected to hydrolysis with concentrated acid and bio-detoxification (Zhang et al., 2019), and then the resultant hydrolysates were used to evaluate the fermentation efficiency of strain A31Z. During the process of simultaneous saccharification, with the addition of 9.71% (w/w) of cellulase, the content of glucose increased along with the process of pretreated products fermentation. However, the concentration of xylose remained unchanged. It was likely that most of xylan had already been hydrolyzed into xylose or oligo-xylan during the pretreatment step, which might lead to the release of the maximum level of xylose in the medium at this point resulting in the failure of further increase in the subsequent saccharification. After 12 h, the concentration of free glucose and xylose in the medium achieved 77.08 g/L and 35.95 g/L, respectively. During the following co-fermentation stage, the conversion of free glucose into ethanol occurred within 24 h after the strain A31Z was inoculated, and then the cells continued to utilize glucose released from cellulose hydrolysis and xylose. In the presence of 15 mg cellulase per gram of dry wheat straw, strain A31Z could produce 56.68 g/L ethanol with a 63.13% sugar-to-alcohol conversion rate (Figure 3C). Approximately, partial ethanol of this conversion came from the initial free glucose released from the pretreatment of hydrolysis, and the rest was from xylose and the glucose released during the SSCF (Liu et al., 2018).

### Fermentation of A31Z in Hydrolysate From Enzymatic Hydrolysis of Engineered *Miscanthus*, Maize, and Wheat Straw

**FIGURE 4:**
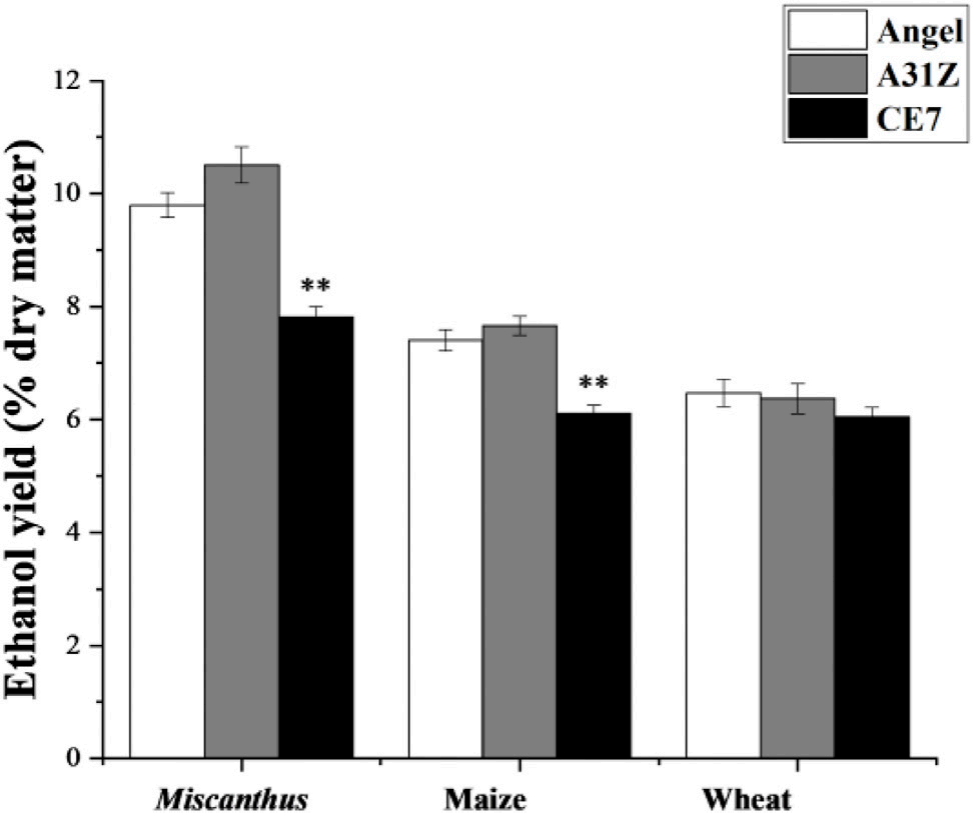
Time-dependent ethanol yield (% dry matter) using *Miscanthus*, maize, and wheat straw as feedstock with strains Angel, A31Z, and CE7. All differences are statistically significant (***p < 0.001; **p < 0.01; *p < 0.05).

Lignocellulosic biomasses such as *Miscanthus*, maize, and wheat straw were the major feedstocks for 2G bioethanol. Hydrolysates from *Miscanthus*, maize, and wheat straw were used as the substrates for ethanol production. As shown in Figure 4, the commercial strain Angel from Wuhan and one of our previous reported strains CE7 were used as controls in this study for the evaluation of the fermentation performance of A31Z at 37°C (Zhang et al., 2019). It was shown that strain A31Z exhibited an ethanol yield of 10.51% of dry matter, which was slightly higher than that of Angel (9.80%) and CE7 (7.82%) from the hydrolysate of *Miscanthus*, while no difference from hydrolysates of maize and wheat straw under the same conditions (Table 1) was observed. However, the xylose utilization of A31Z in the aforementioned three hydrolysates was significantly improved compared to that of the other two strains (Table 2).

**TABLE 1:** Ethanol yield (% dry matter) and concentration (g/L) of different yeast using the hydrolysis of *Miscanthus*, maize, and wheat straw.

Strain	Hydrolysis								
	*Miscanthus*			Maize			Wheat straw		
Ethanol yield (% dry matter)									
Angel	9.80	±	0.22	7.41	±	0.19	6.47	±	0.24
CE7	7.82	±	0.19	6.11	±	0.15	6.05	±	0.16
A31Z	10.51	±	0.32	7.66	±	0.17	6.37	±	0.27
Ethanol concentration (g/L)									
Angel	4.73	±	0.11	3.57	±	0.09	3.12	±	0.12
CE7	3.77	±	0.09	2.95	±	0.07	2.92	±	0.08
A31Z	5.07	±	0.16	3.70	±	0.08	3.07	±	0.13

**TABLE 2:** Sugar-ethanol conversion rate (%) and pentose utilization rate (%) of different yeast using the hydrolysis of *Miscanthus*, maize, and wheat straw.

Strain	Hydrolysis								
	*Miscanthus*			Maize			Wheat straw		
Sugar-to-ethanol conversion rate (%)									
Angel	60.88	±	1.85	66.07	±	2.01	71.26	±	2.17
CE7	56.91	±	1.26	61.76	±	1.37	66.61	±	1.48
A31Z	59.94	±	2.07	65.05	±	2.25	70.16	±	2.42
Pentose utilization rate (%)									
Angel	38.38	±	0.95	22.45	±	1.46	39.65	±	1.19
CE7	28.01	±	1.21	21.15	±	1.43	37.67	±	0.86
A31Z	45.81	±	1.11	34.53	±	1.43	51.86	±	0.89

### Fermentation of A31Z Using Corn and Dried Distillers Grains With Soluble as Feedstocks

**FIGURE 5:**
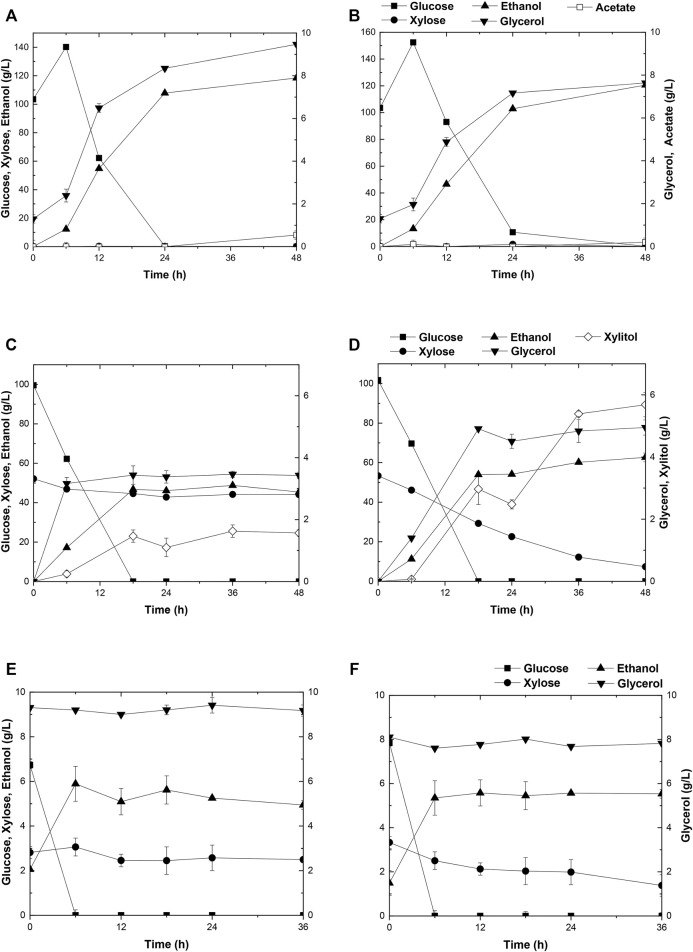
Time-dependent fermentation of Angel and A31Z. Fermentation profile of Angel **(A)** and A31Z **(B)** in corn starch medium. The fermentation profile of Angel **(C)** and A31Z **(D)** in mixed sugar medium of 100 g/L glucose and 50 g/L xylose. The fermentation profile of Angel **(E)** and A31Z **(F)** from DDGS.

The capacity of strain A31Z in first-generation yeast-based ethanol production from corn starch was evaluated. Strain Angel was used as the control. Corn contained approximately two-thirds of starch, and the corn starch was hydrolyzed into glucose prior to fermentation. Strain A31Z could produce 122.32 g/L ethanol, about 2.92 g/L more than that of the control strain after 48 h of fermentation (Figures 5A,B). Besides, its by-product glycerol was about 20% less than that of Angel, which was likely due to the increase in the ethanol production, consistent with a previous study of Scanes et al. (1998) that the level of glycerol was inversely related to that of ethanol (Scanes et al., 1998).

The residues of corn and cells from the 1G ethanol fermentation, known as DDGS, were rich in glucose and xylose and could be used as the feedstock, for further ethanol production. Here, we also evaluated the fermentation performances of Angel and A31Z using a mixture of 100 g/L glucose and 50 g/L xylose as a mimic DDGS hydrolysis (Figures 5C,D). Strain A31Z exhibited both notably higher xylose consuming capacity and ethanol production than those of Angel, with 46.21 g/L and 8.06 g/L for their xylose conversions, and 63.33 g/L and 45.98 g/L for the ethanol productions of the two strains, respectively.

When using the DDGS hydrolysate for fermentation, a slightly higher ethanol production was achieved by A31Z. As shown in Figure 5F, strain A31Z produced an ethanol titer of 5.54 g/L from a total sugar of 11.17 g/L (7.84 g/L glucose and 3.33 g/L xylose) feedstock, and strain Angel produced 4.94 g/L ethanol from 9.56 g/L sugar (6.74 g/L glucose and 2.82 g/L xylose) (Figure 5E). Our fermentation results showed that strain A31Z with a slightly higher ethanol production is superior to strain Angel.

### Simultaneous Saccharification and Co-fermentation of Integrated 1G and 2G Feedstock for Ethanol Production Using A31Z

**FIGURE 6:**
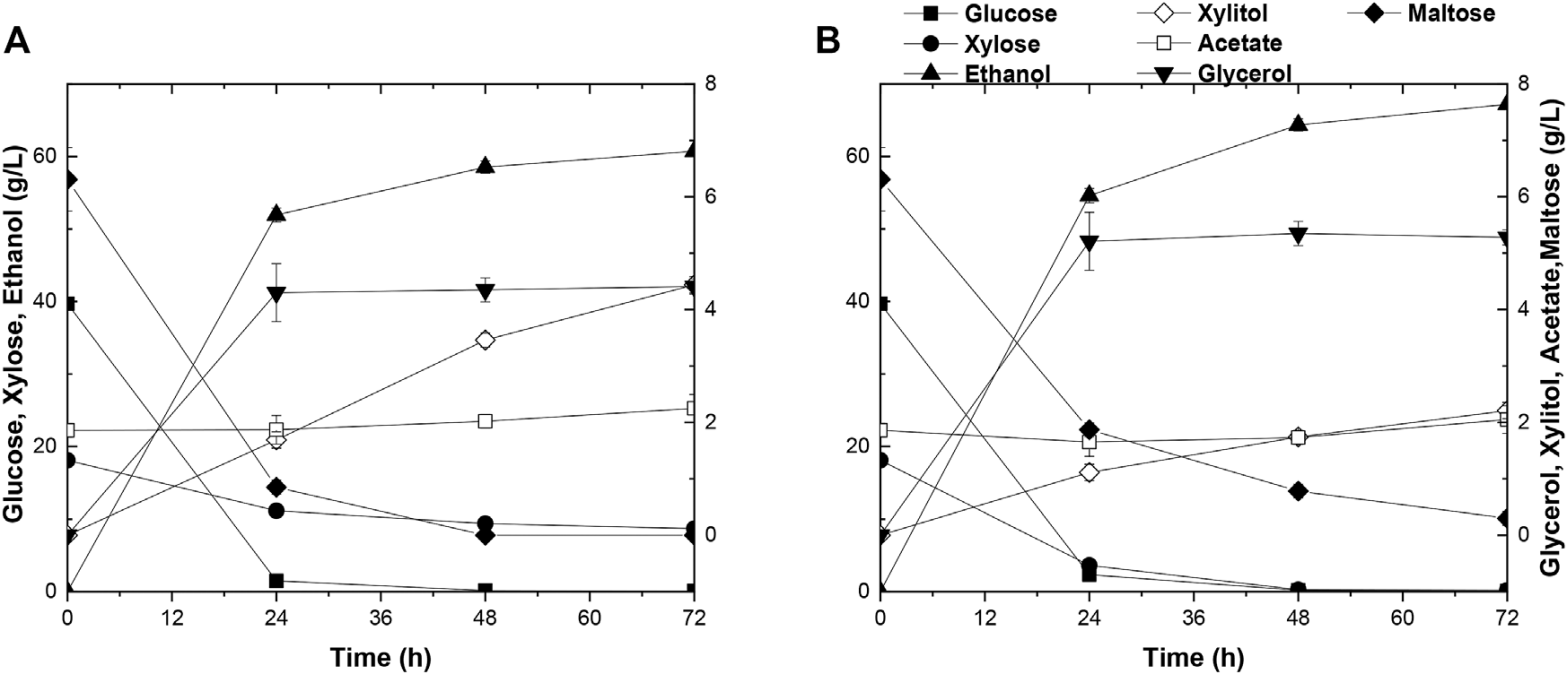
Time-dependent fermentation of Angel and A31Z. Fermentation profile of Angel **(A)** and A31Z **(B)** in SSCF experiments. Dilute acid pretreatment of corn straw with 24% dry matter concentration and equal mass mixture of corn starch with 24% dry matter concentration were used as substrates for yeast fermentation.

As mentioned before, the evolved strain A31Z had shown its excellence in fermentation using various types of substrates. It had been reported that the integrative utilization of whole corn for fermentation could results in a higher ethanol titer and accelerates the application of the 2G technology in ethanol industry (Erdei et al., 2013; Kim et al., 2017). The SSCF data in this study showed that there was a significant improvement in xylose utilization and ethanol production in strain A31Z using the integrated carbon sources of 1G + 2G feedstocks for fermentation (Figures 6A,B). Both strains, Angle and A31Z, consumed glucose completely within 24 h, and the xylose consumption were shown to be 9.42 g/L and 17.93 g/L with ethanol productions of 60.73 g/L and 67.18 g/L within 72 h, respectively. It had been proved that the application of integrative 1G and 2G feedstocks for ethanol industrial production yielded a better economic profit than the standalone of 1G or 2G plant (Dias et al., 2012).

## Discussion

The capacity of monosaccharide utilization is crucial for the yield of ethanol production using lignocellulose biomasses as fermentation feedstock by yeasts. This study provided a roadmap on how to improve cellulosic ethanol production in a diploid yeast strain by progressively optimizing the xylose metabolic pathway via the combination of metabolic genetic engineering and adaptive evolution. Our previous studies demonstrated that imbalanced expression of the set of xylose metabolic enzymes and/or their activities might lead to the disproportion of xylose metabolism in yeasts, thus increasing the accumulation of massive xylitol, an intermediate product of xylose metabolism, resulting in the reduction of the yield of ethanol eventually (Zhang et al., 2019). The rate-limiting gene *TAL1* was identified to play a key role in the balance of metabolites in the pentose phosphate pathway (Zhang et al., 2019). The elevation of *TAL1* expression against an *XYL1(K270R)-XYL2-XKS1* overexpressed background could promote the xylose metabolism, as well as lessen the glycerol and xylitol accumulation (Cao et al., 2014). The overexpression of the gene *PYK1* could facilitate the of improvement the sugar-to-ethanol conversion ratio (Gruning et al., 2011; Gruning et al., 2014). Moreover, the upregulation of the xylose transporter *MGT05196* resulted in a stronger xylose uptake flux, which consequently led to an improvement in its utilization efficiency (Wang et al., 2015). A previous study by Zhang et al. (2019) revealed constitutive promoters of genes in the Embden–Meyerhof–Parnas (EMP) pathway, the tricarboxylic acid cycle (TCA), and stress–response gene family were involved in driving the expression of the xylose metabolic gene cluster in a haploid evolved strain CE7, which accelerated the consumption rate of xylose, and promoted subsequent ethanol production (Zhang et al., 2019). These promoters, involved in different fermentation stages (glucose and xylose stage), played a key role in the xylose metabolism, especially in the presence of acetate (Cao et al., 2014). Kim et al. (2013) constructed an efficient xylose-fermenting diploid yeast strain by mating two engineered haploid yeasts capable of xylose assimilation (Kim et al., 2013). And, it was believed that diploid yeasts were more suitable for the fermentation of industrial hydrolysates (Kim et al., 2013).

All of these previous publications were summarized, which gives a clue to construct a yeast strain via combining the metabolic engineering and adaptive evolution for an improvement of the growth and consequent 2G ethanol production from xylose. The ethanol production in this study was improved from 53.7 to 87.4% of the theoretical yield (from ABN to A21Z), and the obtained yeast A21Z was subjected to genetic modification and evolutionary adaptation. In our work, a highly efficient and stable xylose metabolic pathway was constructed in diploid yeast, paving the path for the 2G bioethanol production.

The numerous inhibitors presented in the lignocellulose hydrolysate created a barrier for yeast fermentation as they severely restricted cell growth. A biorefining approach starting from dry acid pretreatment, disk milling, and biodetoxification of lignocellulose feedstock was employed to counteract the inhibitory effects that occurred in the process of SSCF (Liu et al., 2018). The anti-inhibitory properties of the adaptive strain A31Z manifested in hydrolysates of *Miscanthus*, DDGS, and whole corn could be attributed to the accumulated favorable mutations in its genome. Three strains produced more ethanol yield and volume concentration in the hydrolysate of *Miscanthus* than the other two materials maize and wheat straw; this, in turn, suggests that the hydrolysis of *Miscanthus* can release more mixed glucose and xylose. In *Miscanthus* hydrolysate, A31Z can convert more xylose and glucose into ethanol compared to Angel, indicating that it has better fermentation performance. When total sugars are relatively high, the conversion of sugar to alcohol may differ little, even though ethanol is slightly higher. It would be beneficial in the future to evaluate the contribution of potential target mutations in A31Z by genome sequencing and genetic engineering, and novel gene variants which could be discovered that are responsible for the functional variations related to stress responses, acetate metabolism, and detoxification. Since multiple genes were rationally introduced and integrated in the genome of A31Z, gene expression analysis is required to quantitatively identify the expression ratio of these genes in the PPP, xylose utilization, and transport pathways. Understanding this, it may reveal the quantitative contribution of each enzyme for yeast fermentation on xylose. In this study, 56.7 g/L ethanol was produced by evolved strain A31Z with an overall yield of 63.1% from cellulose and xylose using wheat straw as the feedstock, and a higher xylose conversion ratio (84.9%) was achieved. Higher xylose conversion efficiency in SSCF fermentation accompanied by the generation of minimum amount of wastewater suggested that A31Z was superior to its ancestors.

In addition, our study demonstrated that a xylose-consuming strain for fermentation with hydrolysates from 1G/2G feedstocks was developed, laying the foundation for academic research and industrial large-scale application.

## Data Availability

The original contributions presented in the study are included in the article/Supplementary Material, further inquiries can be directed to the corresponding authors.
