# Supplementary material for: Metabolic and Evolutionary Engineering of Diploid Yeast for the Production of First- and Second-Generation Ethanol
Source: Front Bioeng Biotechnol. 2022 Jan 28;9:835928. doi: 10.3389/fbioe.2021.835928 (PMC8831863; doi:10.3389/fbioe.2021.835928)
Supplement: Supplementary file 1 [file DataSheet1.docx]

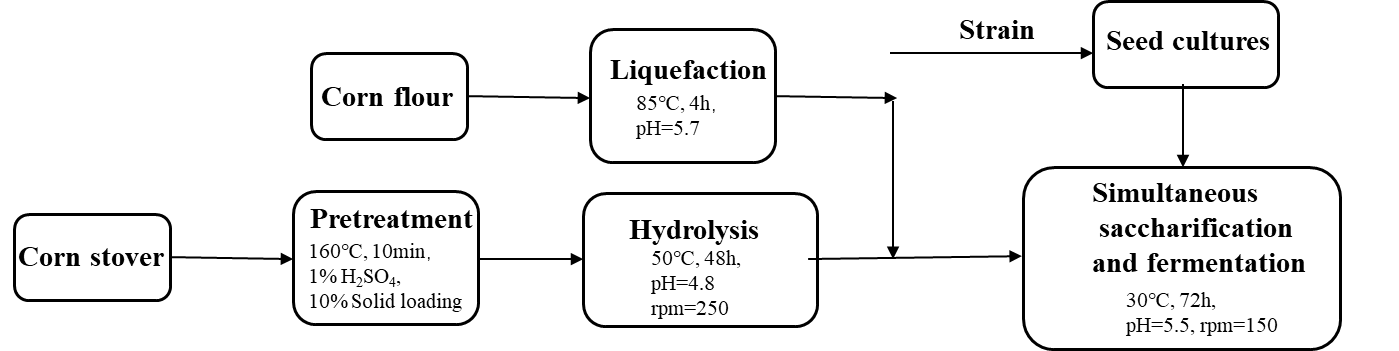


**Supplementary Figure 1.** Illustration of the process used to assess SSCF of sulfuric acid pretreated corn stover and liquefied corn flour in an integrated process configuration.

**Supplementary Table 1**

**Strains used in this study**

| **Name** | **Description** | **References** |
| --- | --- | --- |
| *S. cerevisiae* Angel | Angel yeast | Angel Yeast Co., Ltd China |
| *S. cerevisiae* Henderson | Henderson yeast | Angel Yeast Co., Ltd, China |
| *S. cerevisiae* CE7 | An evolved strain from CE6 | (Zhang et al., 2019) |
| *S. cerevisiae* WXY74 | WXY70, ABA::T1-Z1, T2-Z2, T3-Z3, T4-Z4 | (Zhang et al., 2019) |
| *S. cerevisiae* ABN | Angel, KanMX :: PDC1p*-TKL1*-TKL1t*/*PGK1p*-TAL1*-TAL1t*/*TPI1p*-RKI1*-RKI1t*/*ADH1p*-RPE1*-RPE1t | This study |
| *S. cerevisiae* BBN | Henderson, NAT::ADH1p-*XYL1*(K270R)-ADH1t/PGK1p-*XYL2*-PGK1t/PGK1p-*XKS1*-PGK1t | This study |
| A1 | An evolved strain from ABN | This study |
| A2 | An evolved strain from A1 | This study |
| A21Z  A22Z  A31Z | A2, hygB:: T1-Z1, T2-Z2, T3-Z3, T4-Z4  A2, hygB::T1-Z1, T2-Z2, T3-Z3, T4-Z4 (KL)  An evolved strain from A21Z | This study  This study  This study |

**Supplementary Table 2**

Sugar yields (% dry matter) released from enzymatic hydrolysis of *Miscanthus,* maize and wheat straw with different solid-liquid ratio fermentation

**Supplementary Table 3**

Cell wall composition (% dry matter) from enzymatic hydrolysis of *Miscanthus, maize and wheat straw* determined by a previous described method (Zahoor et al., 2017)


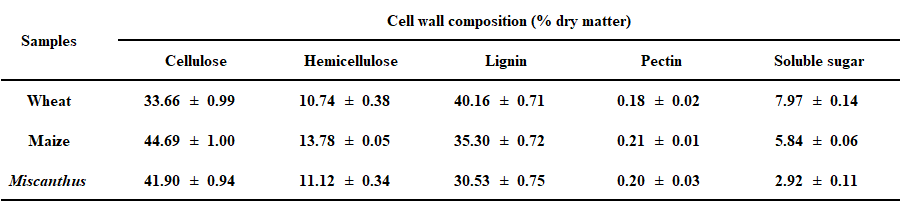


**References**

Zahoor, Tu, Y., Wang, L., Xia, T., Sun, D., Zhou, S., Wang, Y., Li, Y., Zhang, H., Zhang, T., Madadi, M., Peng, L. (2017). Mild chemical pretreatments are sufficient for complete saccharification of steam-exploded residues and high ethanol production in desirable wheat accessions. *Bioresour. Technol.* 243, 319-326. doi:10.1016/j.biortech.2017.06.111

Zhang, C., Xue, Q., Hou, J., Mohsin, A., Zhang, M., Guo, M., Zhu, Y., Bao, J., Wang, J., Xiao, W., Cao, L. (2019). In-depth two-stage transcriptional reprogramming and evolutionary engineering of *Saccharomyces cerevisiae* for efficient bioethanol production from xylose with acetate. *J. Agric. Food. Chem.* 67 (43), 12002-12012. doi:10.1021/acs.jafc.9b05095
